# Supplementary material for: Enriched dietary saturated fatty acids induce trained immunity via ceramide production that enhances severity of endotoxemia and clearance of infection
Source: eLife. 2022 Oct 20;11:e76744. doi: 10.7554/eLife.76744 (PMC9642993; doi:10.7554/eLife.76744)
Supplement: Supplementary file 1. [file elife-76744-supp1.docx]

**Supplemental Table 1. Diet compositions (values represent percentage of total kcal).**

| **Diet** | **Envigo Cat. No.** | **Fat %** | **Carb %** | **Protein %** | **kcal/g** |
| --- | --- | --- | --- | --- | --- |
| Standard Chow | TD.08485 | 13 (3% PA) | 67.9 | 19.1 | 3.6 |
| Western Diet | TD.88137 | 42 (12% PA) | 42.7 | 15.2 | 4.5 |
| Ketogenic Diet | TD.180423 | 90.5 (23% PA) | 0.4 | 9.1 | 6.8 |
| PicoLab Mouse Diet 20 (product 5058) | N/A | 21.6 | 55.2 | 23.2 | 4.6 |
