# Supplementary material for: Enriched dietary saturated fatty acids induce trained immunity via ceramide production that enhances severity of endotoxemia and clearance of infection
Source: eLife. 2022 Oct 20;11:e76744. doi: 10.7554/eLife.76744 (PMC9642993; doi:10.7554/eLife.76744)
Supplement: Supplementary file 2. [file elife-76744-supp2.docx]

**Supplemental Table 2. List of primers used in this study.**

| **Primer** | **Sequence F (5′-3′)** | **Sequence R (5′-3′)** |
| --- | --- | --- |
| TNF | GATCGGTCCCCAAAGGGATG | TGGTTTGTGAGTGTGAGGGTC |
| IL-1β | AGCTTCCTTGTGCAAGTGTCT | GACAGCCCAGGTCAAAGGTT |
| IL-6 | TCC AGT TGC CTT CTT GGG AC | AGT CTC CTC TCC GGA CTT GT |
| IL-10 | GTTGCCAAGCCTTATCGGA | ACCTGCTCCACTGCCTTGCT |
| β-actin | CTGTCCCTGTATGCCTCTG | ATGTCACGCACGATTTCC |
