# Supplementary material for: Enriched dietary saturated fatty acids induce trained immunity via ceramide production that enhances severity of endotoxemia and clearance of infection
Source: eLife. 2022 Oct 20;11:e76744. doi: 10.7554/eLife.76744 (PMC9642993; doi:10.7554/eLife.76744)
Supplement: Transparent reporting form [file elife-76744-transrepform1.pdf]

Westbrook Centre
Milton Road
Cambridge CB4 1YG, UK

P 01223 855340
W elifesciences.org
T @elife

eLife's transparent reporting form

We encourage authors to provide detailed information *within their submission* to facilitate the interpretation and replication of experiments. Authors can upload supporting documentation to indicate the use of appropriate reporting guidelines for health-related research (see EQUATOR Network), life science research (see the BioSharing Information Resource), or the ARRIVE guidelines for reporting work involving animal research. Where applicable, authors should refer to any relevant reporting standards documents in this form.

If you have any questions, please consult our Journal Policies and/or contact us: editorial@elifesciences.org.

Sample-size estimation

- You should state whether an appropriate sample size was computed when the study was being designed
- You should state the statistical method of sample size computation and any required assumptions
- If no explicit power analysis was used, you should describe how you decided what sample (replicate) size (number) to use

Please outline where this information can be found within the submission (e.g., sections or figure legends), or explain why this information doesn't apply to your submission:

Due to predicted variability (based on our previously published and unpublished work and power analysis tool), we have calculated that ~5 mice per group is required to show a difference between Standard Chow mice and Western Diet mice [Napier, 2019], thus we'd predicted the same variability in our ketogenic diet and PA-fed mice. We used the SPH analytics Power Analysis Calculator found here: https://www.dssresearch.com/KnowledgeCenter/toolkitcalculators.aspx

Replicates

- You should report how often each experiment was performed
- You should include a definition of biological versus technical replication
- The data obtained should be provided and sufficient information should be provided to indicate the number of independent biological and/or technical replicates
- If you encountered any outliers, you should describe how these were handled
- Criteria for exclusion/inclusion of data should be clearly stated
- High-throughput sequence data should be uploaded before submission, with a private link for reviewers provided (these are available from both GEO and ArrayExpress)

Please outline where this information can be found within the submission (e.g., sections or figure legends), or explain why this information doesn't apply to your submission:

eLife Sciences Publications, Ltd is a limited liability non-profit non-stock corporation incorporated in the State of Delaware, USA, with company number 5030732, and is registered in the UK with company number FC030576 and branch number BR015634 at the address Westbrook Centre, Milton Road, Cambridge CB4 1YG.

Biological replicates are specified groups of independent samples used to account for random biological variation that may occur within the groups. Technical replicates are repeated and identical sample treatments that are used to refine the reproducibility of an assay.

In all *in vitro* experiments we used technical triplicates within each experiment and each experiment was validated in biological triplicates. All *in vivo* experiments we used at least 5 mice per group (each *n* value is reported within the figure legend) and each experiment was run in at least biological triplicates. We kept in all outliers for every *in vitro* and *in vivo* experiment and did not take out any data points for biological or technical replicates.

Please refer to the figure legends for the number of experiments represented in each figure, sample sizes, and biological and technical replicates.

eLife Sciences Publications, Ltd is a limited liability non-profit non-stock corporation incorporated in the State of Delaware, USA, with company number 5030732, and is registered in the UK with company number FC030576 and branch number BR015634 at the address Westbrook Centre, Milton Road, Cambridge CB4 1YG.

Statistical reporting

- Statistical analysis methods should be described and justified
- Raw data should be presented in figures whenever informative to do so (typically when N per group is less than 10)
- For each experiment, you should identify the statistical tests used, exact values of N, definitions of center, methods of multiple test correction, and dispersion and precision measures (e.g., mean, median, SD, SEM, confidence intervals; and, for the major substantive results, a measure of effect size (e.g., Pearson's r, Cohen's d)
- Report exact p-values wherever possible alongside the summary statistics and 95% confidence intervals. These should be reported for all key questions and not only when the p-value is less than 0.05.

Please outline where this information can be found within the submission (e.g., sections or figure legends), or explain why this information doesn't apply to your submission:

Justification for statistical analysis can be found under "Statistics" in Materials and Methods.

Raw data is included in figures when it is informative, however figures become difficult to see for many of the figures due to multiple time points and high *n*'s, so we have included all raw data in supplemental source data files.

We have identified the statistical tests used in each figure legend, included exact values of *n* for in vivo experiments and dispersion and precisions measures.

We have reported exact p values within the supplemental source data files and defined our definition of *, **, ***, **** within each figure legend.

eLife Sciences Publications, Ltd is a limited liability non-profit non-stock corporation incorporated in the State of Delaware, USA, with company number 5030732, and is registered in the UK with company number FC030576 and branch number BR015634 at the address Westbrook Centre, Milton Road, Cambridge CB4 1YG.

(For large datasets, or papers with a very large number of statistical tests, you may upload a single table file with tests, Ns, etc., with reference to sections in the manuscript.)

Group allocation

- Indicate how samples were allocated into experimental groups (in the case of clinical studies, please specify allocation to treatment method); if randomization was used, please also state if restricted randomization was applied
- Indicate if masking was used during group allocation, data collection and/or data analysis

Please outline where this information can be found within the submission (e.g., sections or figure legends), or explain why this information doesn't apply to your submission:

Groups were determined based on their treatments and timepoints. Masking was not used during data collection or data analysis.

Additional data files ("source data")

- We encourage you to upload relevant additional data files, such as numerical data that are represented as a graph in a figure, or as a summary table
- Where provided, these should be in the most useful format, and they can be uploaded as "Source data" files linked to a main figure or table
- Include model definition files including the full list of parameters used
- Include code used for data analysis (e.g., R, MatLab)
- Avoid stating that data files are "available upon request"

Please indicate the figures or tables for which source data files have been provided:

Source data and code files are provided for all figures and statistical analysis is included in each source file.

eLife Sciences Publications, Ltd is a limited liability non-profit non-stock corporation incorporated in the State of Delaware, USA, with company number 5030732, and is registered in the UK with company number FC030576 and branch number BR015634 at the address Westbrook Centre, Milton Road, Cambridge CB4 1YG.
